# Lack of OxyR and KatG Results in Extreme Susceptibility of *Francisella tularensis* LVS to Oxidative Stress and Marked Attenuation *In vivo*

**DOI:** 10.3389/fcimb.2017.00014

**Published:** 2017-01-24

**Authors:** Marie Honn, Helena Lindgren, Gurram K. Bharath, Anders Sjöstedt

**Affiliations:** Clinical Bacteriology, and Laboratory for Molecular Infection Medicine Sweden, Department of Clinical Microbiology, Umeå UniversityUmeå, Sweden

**Keywords:** *Francisella tularensis*, OxyR, KatG, oxidative stress, virulence

## Abstract

*Francisella tularensis* is an intracellular bacterium and as such is expected to encounter a continuous attack by reactive oxygen species (ROS) in its intracellular habitat and efficiently coping with oxidative stress is therefore essential for its survival. The oxidative stress response system of *F. tularensis* is complex and includes multiple antioxidant enzymes and pathways, including the transcriptional regulator OxyR and the H_2_O_2_-decomposing enzyme catalase, encoded by *katG*. The latter is regulated by OxyR. A deletion of either of these genes, however, does not severely compromise the virulence of *F. tularensis* and we hypothesized that if the bacterium would be deficient of both catalase and OxyR, then the oxidative defense and virulence of *F. tularensis* would become severely hampered. To test this hypothesis, we generated a double deletion mutant, Δ*oxyR/*Δ*katG*, of *F. tularensis* LVS and compared its phenotype to the parental LVS strain and the corresponding single deletion mutants. In accordance with the hypothesis, Δ*oxyR/*Δ*katG* was distinctly more susceptible than Δ*oxyR* and Δ*katG* to H_2_O_2_, ONOO^−^, and ${\text{O}}_{2}^{-}$, moreover, it hardly grew in mouse-derived BMDM or in mice, whereas Δ*katG* and Δ*oxyR* grew as well as *F. tularensis* LVS in BMDM and exhibited only slight attenuation in mice. Altogether, the results demonstrate the importance of catalase and OxyR for a robust oxidative stress defense system and that they act cooperatively. The lack of both functions render *F. tularensis* severely crippled to handle oxidative stress and also much attenuated for intracellular growth and virulence.

## Introduction

*Francisella tularensis*, a Tier 1 select agent and the causative agent of tularemia, is a zoonotic, facultative intracellular bacterium with two clinically relevent subspecies, *tularensis* and *holarctica*, the former of which causes an aggressive disease with high mortality if left untreated (Oyston et al., 2004). Although there is no licensed vaccine against this potential bioterrorism agent, the subspecies *holarctica* live vaccine strain, LVS, is used to vaccinate laboratory workers, and is widely used in *Francisella* research as it is attenuated in humans, but retains its virulence in mice (Sjöstedt, 2006; Conlan, 2011).

*Francisella tularensis* is capable of infecting numerous cell types, including professional phagocytes, like macrophages. Upon phagocytosis, it transiently resides within the phagosome before escaping into the cytosol to replicate (Bröms et al., 2010; Chong and Celli, 2010). Phagocytes constitute a hostile environment utilizing a wide array of anti-bacterial mechanisms, such as phagosome acidification, disruption of pathogen membrane integrity, removal or sequestration of nutrients, and the production of reactive oxygen species (ROS) (Flannagan et al., 2009) and since *F. tularensis* is an intracellular bacterium, it will encounter a continuous exposure to ROS. Vital macromolecules, such as proteins and DNA, will react with ROS, thereby disrupting their functions (Fridovich, 1998; Schaible and Kaufmann, 2004; Flannagan et al., 2009). There are several ROS with potent antibacterial effects, such as superoxide and H_2_O_2_. The former is produced at high levels by the phagocyte oxidase (phox) and it rapidly combines with nitric oxide (NO), which is produced at high levels by inducible nitric oxide synthase (iNOS), to form peroxynitrite, a highly reactive compound. H_2_O_2_ is toxic *per se*, but the damage it exerts can be exacerbated in combination with intracellular ferrous iron, resulting in the formation of hydroxyl radicals (HO^•^) and hydroxide anions (OH^−^) through the Fenton reaction.

Reactive oxygen species (ROS) are not only formed during host attack, but low levels are also formed as by-products of normal aerobic metabolism. Thus, pathogens, in particular intracellular pathogens, have a pressing need for defense mechanisms to combat the ever present levels of ROS, but even more so to combat the assault of ROS experienced within a host (Betteridge, 2000). The critical roles of ROS and NO for the host defense against tularemia are illustrated by the extreme susceptibility of phox-deficient and iNOS-deficient mice to an *F. tularensis* infection (Lindgren et al., 2004). Moreover, *ex vivo*, it has been demonstrated that the requirements for host protection vary depending on the cell type investigated, since killing of *F. tularensis* by mouse peritoneal cells is NO-dependent, but NO-independent by mouse pulmonary cells (Anthony et al., 1992; Polsinelli et al., 1994; Lindgren et al., 2005).

The oxidative stress defense system of *Escherichia coli* has been extensively studied and includes numerous detoxifying enzymes, such as catalase, superoxide dismutases (SODs), alkyl hydroperoxide reductase (Ahp), and the H_2_O_2_-activated transcriptional regulator OxyR. The latter combats the effect of H_2_O_2_ by dual mechanisms, since it regulates the expression of both catalase and the ferric uptake regulator (Fur) (Farr and Kogoma, 1991; Zheng et al., 1998, 1999; Pomposiello and Demple, 2001). Catalase renders H_2_O_2_ harmless by degrading it to oxygen and water, whilst Fur down-regulates the expression of genes involved in iron uptake, thus limiting the amount of iron with which H_2_O_2_ can combine in the Fenton reaction (Andrews et al., 2003; Troxell and Hassan, 2013). Catalase, SODs, AhpC and other detoxifying enzymes are employed as oxidative stress defense mechanisms also by *F. tularensis* (Bakshi et al., 2006; Lindgren et al., 2007; Melillo et al., 2009; Binesse et al., 2015). The *F. tularensis* catalase, encoded by *katG*, mediates H_2_O_2_ tolerance and is known to be important for the virulence of *F. tularensis* LVS (Lindgren et al., 2007). SodB, FeSOD, and SodC, CuZnSOD, are both known to be important for the dismutation of ${\text{O}}_{2}^{-}$ in *F. tularensis*, and SodB further acts in the defense against oxidative stress by harnessing iron (Bakshi et al., 2006; Melillo et al., 2009). The *F. tularensis* AhpC enzyme is important for the detoxification of ${\text{O}}_{2}^{-}$ and peroxynitrite (ONOO^−^), but not of H_2_O_2_, in the highly virulent SCHU S4 strain (Binesse et al., 2015), but the importance in the LVS strain is yet unknown. *F. tularensis* also encodes an *oxyR* homolog, the role of which has been studied recently (Ma et al., 2016). It was found that the absence of OxyR rendered LVS defective for oxidative stress defense, growth in macrophages and epithelial cells, and virulence in mice. Moreover, it was demonstrated that OxyR regulates the expression of the *ahpC, katG*, and *sodB* genes, with the most pronounced regulatory effect exerted on *ahpC*.

A more thorough understanding of the *F. tularensis* antioxidant system will undoubtedly reveal virulence mechanisms of this bacterium, since ROS constitute such an essential threat to the pathogen. As aforementioned, antioxidant enzymes, such as catalase, AhpC, SodC, and SodB, all contribute to the virulence of *F. tularensis* in mice, although each appears to render the bacterium only moderately attenuated and this indicates that the antioxidant system of *F. tularensis* is complex and may in part possess overlapping functions (Lindgren et al., 2007; Ma et al., 2016). Indeed, a double deletion mutant of *katG* and *ahpC* has not been possible to generate in *F. tularensis* (Binesse et al., 2015) and this demonstrates that the cooperative functions of these enzymes are crucial, although either one is not essential. The aim of the present study was to better understand this interconnecting web of antioxidants in *F. tularensis*. To this end, a double deletion mutant, Δ*oxyR/*Δ*katG*, was generated since this mutant, besides lack of catalase activity, should have a repressed expression of OxyR-regulated antioxidant genes, one of which is AhpC (Ma et al., 2016). We hypothesized that the lack of both KatG and OxyR would lead to a severely impaired phenotype of *F. tularensis* LVS. We therefore characterized the phenotypes of single deletion mutants, Δ*oxyR* and Δ*katG*, and a double deletion mutant, Δ*oxyR/*Δ*katG*, in comparison to the parental LVS strain.

## Materials and methods

### Bacterial strains

The *F. tularensis* LVS strain was obtained from the *Francisella* strain collection (FSC) at FOI, Swedish Defense Research Agency. The *katG* deletion mutant (Δ*katG*) has been described previously (Lindgren et al., 2007).

The Δ*oxyR* and Δ*oxyR/*Δ*katG* mutants of the LVS strain were generated by allelic replacement as described previously (Golovliov et al., 2003). Briefly, sequences up- and down-stream of *oxyR* were amplified by PCR. The fragments contained complementary sequences, which were joined together by a second PCR. The resulting fragment was cloned into the pDM4 suicide-vector, which was transformed into *Escherichia coli* S17-λpir and thereafter transferred to LVS by conjugation. Clones with a successful recombination event were selected on plates supplemented with Cm and polymyxin B. Correct integration was confirmed by PCR. Positive clones were subjected to sucrose selection to select for a second recombination event and clones were screened by PCR to identify successful deletion mutants. The double deletion mutant Δ*oxyR/*Δ*katG* was generated using the same procedure, apart from using the pDMK3 plasmid carrying kanamycin resistance. The deletions were verified by sequencing 1500 bp on each side of the deleted region.

### Aerobic and microaerobic growth

Bacteria were cultivated overnight on plates based on modified GC-agar (MC plates) and then inoculated to an OD_600_ of 0.1 in Chamberlain's chemically defined medium (CDM). All cultures were split into triplicates and were incubated at 37°C and 200 rpm in an aerobic (normal air) or a microaerobic (10% O_2_ and 10% CO_2_) milieu up to 48 h with monitoring of the OD_600_.

### H_2_O_2_ susceptibility assay

Bacteria were cultivated overnight on MC plates, inoculated to an OD_600_ of 0.1 in CDM and H_2_O_2_ was added to the final concentration of 0.02, 0.1, or 0.5 mM, respectively. Controls were grown without the addition of H_2_O_2_. All cultures were split into triplicates and were incubated at 37°C and 200 rpm up to 24 h with monitoring of the OD_600_.

### Catalase activity assay

Catalase degrades H_2_O_2_ to O_2_ and H_2_O. H_2_O_2_ absorbs light at 240 nm and degradation of H_2_O_2_ can therefore be measured as a reduction of A_240_ nm over time.

Strains were cultivated overnight after being diluted to an OD_600_ of 0.1 in CDM. For each strain, one set of tubes were left untreated and another set of tubes were supplemented with H_2_O_2_ to a final concentration of 0.02, 0.1, or 0.2 mM. All cultures were split into triplicates and incubated at 37°C, 200 rpm for 2, 4, and 24 h before sampling for evaluation of catalase activity. Depending on the density and growth phase of the culture, a volume of 10–50 μl were withdrawn and diluted in PBS to reach a final volume of 120 μl in UV-clear 96-well plates (Greiner Bio-one, Frickenhausen, Germany). Then, 80 μl 100 mM H_2_O_2_ in PBS was added to each sample immediately before placing the plate in a Tecan Infinite 200 pro plate reader and measuring the reduction in absorption at 240 nm for 10 min. A molar extinction coefficient of H_2_O_2_ at 240 nm of 43.6 M^−1^cm^−1^ was used to calculated the concentration of H_2_O_2_ using the Beer-Lambert law, *A* = ε*cl*. One unit of catalase is defined as the amount that decomposes 1 μmol of H_2_O_2_ per minute per OD_600_ at 25°C. The catalase units were normalized against the OD of the culture.

### Paraquat susceptibility assay

Susceptibility of *F. tularensis* strains to ${\text{O}}_{2}^{-}$ was determined by use of the ${\text{O}}_{2}^{-}$ generating compound paraquat dichloride hydrate (Sigma-Aldrich, St. Louis, USA) in a disc diffusion assay. Paraquat generates ${\text{O}}_{2}^{-}$ through reacting with parts of the respiratory chain in bacteria, causing the reduction of O_2_ to ${\text{O}}_{2}^{-}$ (Hassan and Fridovich, 1979). Bacterial strains were cultivated on MC plates overnight, re-suspended in phosphate-buffered saline (PBS) and approximately 3 × 10^5^ CFU were plated onto MC plates. Sterile filter discs (Oxoid Blank Antimicrobial Susceptibility Discs, Thermo Scientific, MA, USA) were placed in the center the plates once they had dried, and 10 μl of MQ-water, 1.25 mM, 5 mM or 20 mM paraquat solution was added to each disc. The plates were incubated for 4 days at 37°C, 5% CO_2_ before the size of the growth inhibition zone surrounding each disc was determined.

### Peroxynitrite susceptibility assay

3-morpholinosydnonimine hydrochloride (SIN-1) (Molecular Probes, Oregon, USA) spontaneously releases (NO) and ${\text{O}}_{2}^{-}$ under physiological conditions, thereby generating peroxynitrite (ONOO^−^). Under physiological conditions 1 mM SIN-1 generates 10 μM ONOO^−^/min (Lindgren et al., 2007).

Strains were cultivated in CDM to logarithmic growth phase and diluted to a density of approximately 2 × 10^6^ bacteria/ml in PBS. The bacterial suspensions were incubated with or without the addition of 0.48 mM SIN-1 with equal amounts of SIN-1 added at the start of the experiment and again after 1.5 h to ensure stable levels of ONOO^−^ (Lindgren et al., 2005). After 3 h samples were collected, diluted and plated on MC plates for determination of viable bacteria.

### Analysis of gene expression by real time PCR

Bacteria were cultivated overnight on MC plates, inoculated to an OD_600_ of 0.1 in CDM and incubated at 37°C, 5% CO_2_ for 10 h before sampling. RNA extraction, cDNA synthesis and Real Time PCR (RT-PCR) were all performed as described previously (Honn et al., 2012).

Briefly, RNA was extracted using Trizol reagent (Invitrogen, CA, USA) from pelleted bacteria, 3 × 10^9^ CFU/sample. Contaminating DNA was removed using the DNA-free kit (Ambion, Inc, Austin, TX, USA) and RNA was quantified by Nanodrop (Thermo Fisher Scientific, Wilmington, DE, USA). cDNA was synthesized from 1 μg RNA/sample using iScript (BioRad, Hemel, Hampstead, UK), RT-PCR was performed using the *Power* SYBR green PCR Master Mix (Applied Biosystems, Foster City, CA, USA) and the ABI Prism 7900Ht Sequence Detection System (Applied Biosystems) as described (Honn et al., 2012). Trizol, DNA-free, iScript and Power SYBR green were all used in accordance with the instructions provided by the manufacturers. Forward and reverse primers were obtained from Invitrogen and have been published previously for *fslA* (*FTL_1832*), *fslB* (*FTL_1833*), *fslC* (*FTL_1834*), *fslD* (*FTL_1835*), *fslE* (*FTL_1836*), *fupA* (*FTL_0439*), *furA* (*FTL_1831*), (Lindgren et al., 2009), *tul4, iglC* (*FTL_0113*), (Bröms et al., 2009), *mglA* (*FTL_0260*), *feoB* (*FTL_0133*), and *katG* (*FTL_1504*) (Honn et al., 2012), sequences for, *grxA* (*FTL_0985*), *grxB* (*FTL_1792*), *gpx* (*FTL_1383*), *sspA* (*FTL_1606*), *ahpC1* (*FTL_0542*), *ahpC2* (*FTL_1191*), *sodB* (*FTL_0380*), *sodC* (*FTL_1791*), *clpB* (*FTL_0094*), *groES* (*FTL_1715*), *groEL* (*FTL_1714*), and *dnaK* (*FTL_1191*) are available upon request.

The Ct values of the selected genes were normalized to the Ct value of the house keeping gene *FTT0901* (*lpnA*) and relative copy numbers (RCN) were calculated according to the following equation: RCN = 2^−ΔCt^ × 100, where ΔCt is Ct(target)−Ct(*FTT0901*) (Gavrilin et al., 2006). Thus, the copy number of a given gene is related to the copy number of *FTT0901*. Normalized Ct values were used for statistical evaluation of the data by One way ANOVA followed by Tukey's honest significant difference (HSD).

### Preparation and infection of BMDM

The capacity of LVS and the mutants to proliferate intracellularly were assessed in bone marrow-derived macrophages (BMDMs). BMDMs were generated from C57BL/6 mice essentially as described previously (Bröms et al., 2011).

The day before infection, BMDM cells were seeded at a density of 4 × 10^5^ cells/ml in 24-well tissue-culture plates and incubated at 37°C, 5% CO_2_ with or without murine recombinant 1000 U/ml of IFN-γ (Peprotech, Rocky Hill, NJ, USA) The next day, the cells were washed and reconstituted with fresh, pre-warmed culture media. Bacteria were grown overnight on MC plates and re-suspended in PBS to a density of approximately 3 × 10^9^ bacteria/ml. Bacteria were diluted in DMEM and added to each well at multiplicity of infection of 30 and bacterial uptake was allowed to occur for 90 min at 37°C, 5% CO_2_. Remaining extracellular bacteria were removed by rinsing the monolayers three times with DMEM and incubating with gentamicin for 45 min followed by rinsing the monolayers three times. This time-point was defined as 0 h. After 0, 4 and 24 h incubation the macrophages were lysed in 0.1% deoxycholate in PBS. The lysate were serially diluted in PBS and plated on MC plates for determination of viable bacteria.

### Mouse experiments

Virulence of the mutant strains was determined by subcutaneous infection of female C57BL/6 mice with 4 × 10^3^ CFU/mouse of LVS, Δ*oxyR*, Δ*katG*, and Δ*oxyR/*Δ*katG*. Mice were monitored for signs of illness and were euthanized by inhalation of isoflurane followed by CO_2_ asphyxiation after 3 or 6 days, whereupon the number of viable bacteria in spleens and livers were determined by homogenizing the organs in PBS and plating dilutions on MC plates. All animal experiments were approved by the Local Ethical Committee on Laboratory Animals, Umeå, Sweden (no. A 1-09, A 99-11, and A 67-14).

### Statistical analysis

One way ANOVA followed by Tukey's HSD test was used to determine statistical significant difference between groups.

## Results

### Growth under aerobic vs. microaerobic conditions

**Figure 1:**
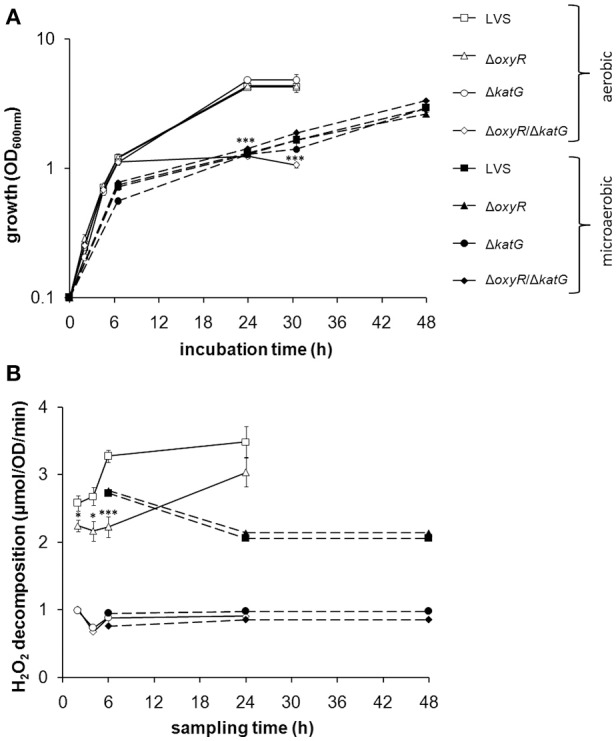
**(A)** Growth of *F. tularensis* strains in CDM under aerobic or microaerobic conditions and **(B)** catalase activity of the strains at indicated time-points during growth. **(A)** Shows a representative experiment of at least three performed and **(B)** the average from six to nine separate observations for each time point and growth condition. Error bars represent the SEM. ^*^P < 0.05, ^***^P < 0.001 vs. LVS.

CDM effectively supports growth of LVS. We therefore compared growth of the bacterial strains, LVS, Δ*oxyR*, Δ*katG*, and Δ*oxyR/*Δ*katG*. The former three strains all replicated to the same extent, whereas Δ*oxyR/*Δ*katG* showed intact growth to late log phase, but impaired growth thereafter. Therefore, it did not reach as high densities as LVS and the other strains at 24 h (*P* < 0.001; Figure 1A). To explore if a reduced oxygen tension could rescue the growth of Δ*oxyR/*Δ*katG*, the strains were cultivated under microaerobic conditions, i.e., 10% O_2_ and 10% CO_2_. Indeed, Δ*oxyR/*Δ*katG* grew as well as the other strains and reached an optical density of > 2.0 within 48 h (Figure 1A). As noted before (Honn et al., 2012), the growth rate of LVS under microaerobic conditions was reduced compared to aerobic conditions (Figure 1A).

### Catalase activity under aerobic vs. microaerobic conditions

The results so far suggested that LVS experienced oxidative stress during growth in an aerobic environment and to handle this stress, required either the function of catalase, or the expression of OxyR-regulated detoxifying mechanisms. OxyR is known to respond to oxidative stress by inducing antioxidant enzymes, such as catalase. As an indicator of oxidative stress and to investigate if catalase is under the regulation of *oxy*R in LVS, we measured the activity of the enzyme during growth of the bacteria in CDM. The catalase activity in LVS gradually increased during the two to 24 h period, whereas the catalase activity in Δ*oxyR* was sustained at a constant, but lower level compared to LVS from two to six h (*P* < 0.05 at 2 and 4 h and *P* < 0.001 at 6 h; Figure 1B). However, the catalase activity of the two strains was similar at 24 h (Figure 1B). In the microaerobic environment, the catalase activity of LVS and Δ*oxyR* was similar, but for both lower than in the aerobic environment (Figure 1B). The H_2_O_2_ decomposition in samples containing Δ*katG* or Δ*oxyR/*Δ*katG* was below 1 μmol, regardless of growth condition and time point, indicating the absence of catalase activity (Figure 1B).

In summary, Δ*oxyR* demonstrated a basal catalase activity, but did not induce this activity further during the aerobic logarithmic growth phase as LVS did. Δ*oxyR/*Δ*katG*, which lacks this basal catalase activity, failed to grow to high densities under the aerobic condition, but grew as well as LVS in the microaerobic milieu.

### H_2_O_2_ tolerance

**Figure 2:**
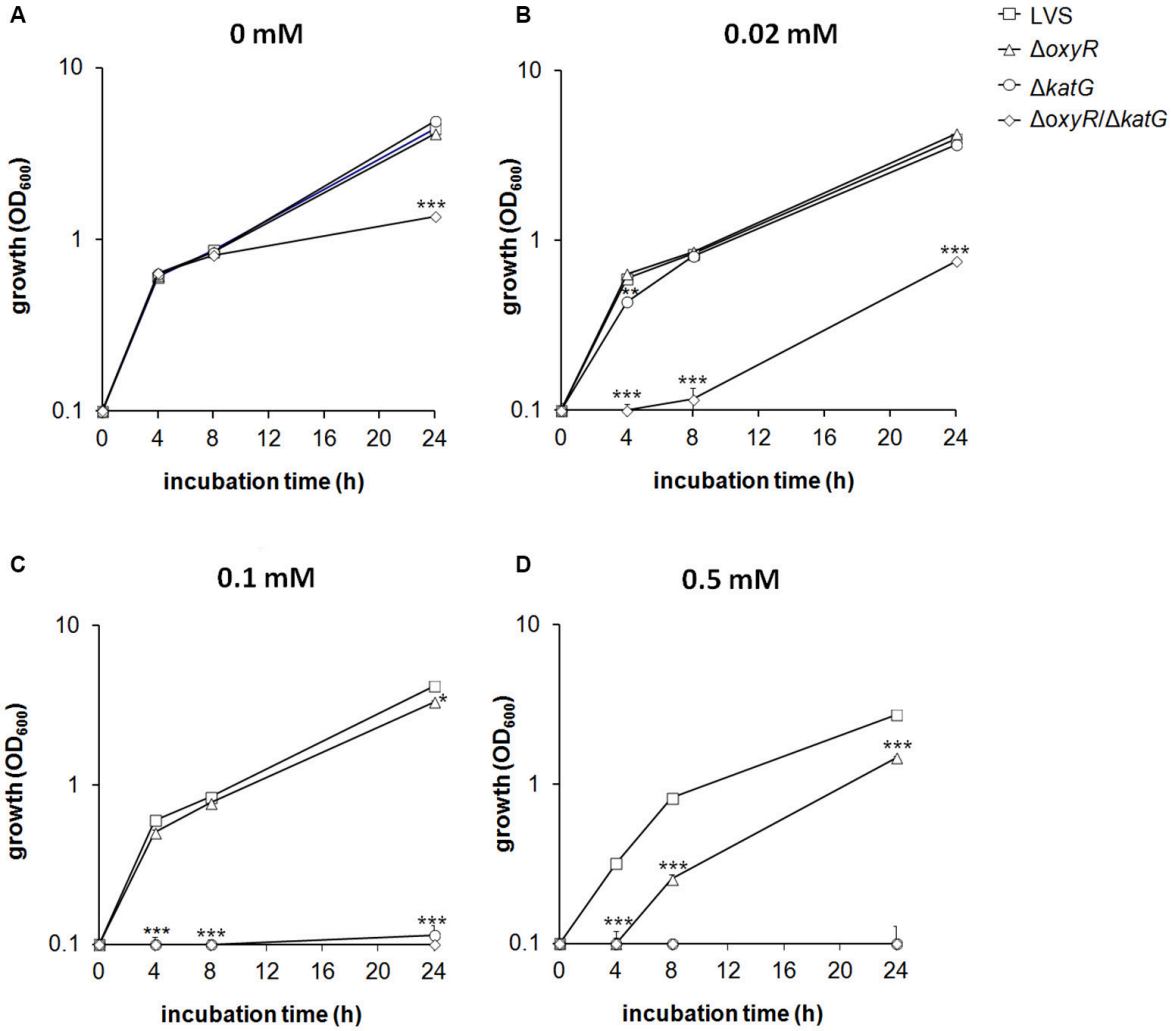
**Growth of *F. tularensis* strains in CDM during exposure to various concentrations of H_2_O_2_ (A)** 0 mM, **(B)** 0.02 mM, **(C)** 0.1 mM, and **(D)** 0.5 mM. The results shown illustrate one representative experiment of at least three performed. Each value represents the average for triplicate samples and error bars represent the SEM. ^*^P < 0.05, ^**^P < 0.01, ^***^P < 0.001 vs. LVS.

Δ*oxyR* and Δ*katG* grew as well as LVS in CDM despite the reduced, or lack of catalase activity (Figure 2A). To investigate their adaptation to stress, H_2_O_2_, the substrate of catalase, was added to the cultures. Growth of LVS or Δ*oxyR* was not affected by 0.02 mM H_2_O_2_, whereas, initially, the growth rate of Δ*katG* was reduced (*P* < 0.01) and growth of Δ*oxyR/*Δ*katG* almost completely inhibited (*P* < 0.001; Figure 2B). At 0.1 mM of H_2_O_2_, LVS and Δ*oxyR* still grew rapidly, in contrast to Δ*katG* and Δ*oxyR/*Δ*katG* that did not grow at all (*P* < 0.001; Figure 2C). Growth of Δ*oxyR* was significantly reduced in the presence of 0.5 mM of H_2_O_2_ compared to LVS (*P* < 0.001; Figure 2D). Exposure of the strains to H_2_O_2_ did not significantly change their catalase activity (data not shown).

In summary, the mutant strains displayed increased susceptibility to H_2_O_2_ as compared to LVS, with the effect being most pronounced for Δ*oxyR/*Δ*katG*, followed by Δ*katG*, and the least affected strain being Δ*oxyR*.

### Susceptibility to paraquat-mediated killing

**Figure 3:**
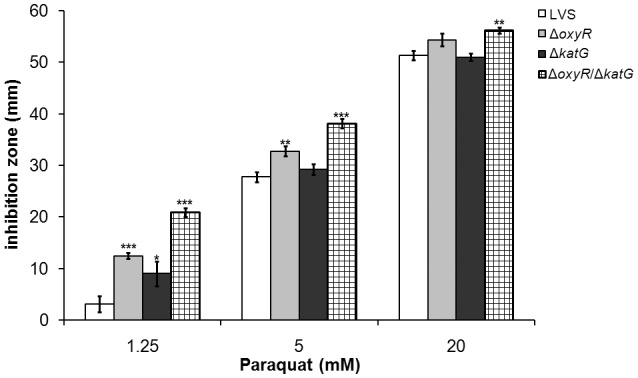
***F. tularensis* strains were exposed to the ${\text{O}}_{2}^{-}$-generating compound paraquat in a disc diffusion assay**. Each bar represents the average from three separate experiments with triplicate samples in each and error bars represent the SEM. ^*^P < 0.05, ^**^P < 0.01, ^***^P < 0.001 vs. LVS for each concentration.

${\text{O}}_{2}^{-}$ is continuously generated as a by-product of the respiratory chain during growth of bacteria. To investigate the capacity of the bacteria to defend against such ROS, LVS, Δ*oxyR*, Δ*katG*, and Δ*oxyR/*Δ*katG* were exposed to paraquat in a disc diffusion assay (Figure 3). Paraquat dichloride hydrate generates ${\text{O}}_{2}^{-}$ through a reaction with parts of the respiratory chain in bacteria, causing the reduction of O_2_ to ${\text{O}}_{2}^{-}$ (Hassan and Fridovich, 1979). Δ*oxyR* displayed a significantly larger zone of inhibition than did LVS in the presence of 1.25 and 5 mM paraquat (*P* < 0.001 and 0.01, respectively), but the zones were similar when exposed to 20 mM (Figure 3). The zone of inhibition for Δ*katG* was larger compared to LVS at 1.25 mM (*P* < 0.05), but similar at the two higher concentrations (Figure 3). A significantly larger zone of inhibition was observed for Δ*oxyR/*Δ*katG* vs. LVS and Δ*katG* at all three concentrations of paraquat (*P* < 0.001 for 1.25 and 5 mM and *P* < 0.01 for 20 mM) and also larger compared to Δ*oxyR* at 1.25 and 5 mM (*P* < 0.01; Figure 3).

In summary, the results demonstrated that Δ*oxyR* and Δ*oxyR/*Δ*katG* were more susceptible to paraquat-mediated killing compared to LVS, with Δ*oxyR/*Δ*katG* being the most susceptible, whereas Δ*katG* was only slightly more susceptible than LVS.

### Susceptibility to SIN-1-mediated killing

Peroxynitrite (ONOO^−^) is a highly reactive and bactericidal ROS formed through the reaction between (NO) and ${\text{O}}_{2}^{-}$ and it is active against *F. tularensis* in activated macrophages (Lindgren et al., 2005). Experimentally, SIN-1 can be used to mimic a continuous exposure to ONOO^−^. SIN-1 slowly decomposes, thereby releasing both NO and ${\text{O}}_{2}^{-}$ that combine to form ONOO^−^, which quickly is internalized since it passes through lipid bilayers (Hogg et al., 1992; Murphy et al., 1998).

**Figure 4:**
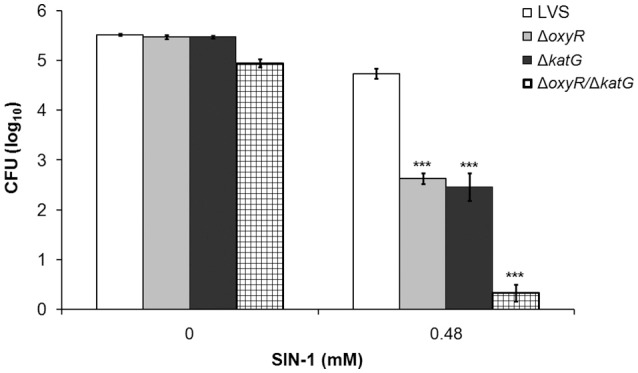
***F. tularensis* strains were exposed to the peroxynitrite generating compound SIN-1 for 3 h**. After 1.5 h of incubation, additional SIN-1 was added to the tubes to ensure a constant generation of peroxynitrite during the whole incubation period. Each bar represents the average from three separate experiments with triplicate samples in each and error bars represent the SEM. ^***^P < 0.001 vs. LVS.

The exposure to 0.48 mM SIN-1 for 3 h reduced the viability of all strains in comparison to un-treated cultures (*P* < 0.001 for all strains), but affected the mutant strains to a greater extent compared to LVS (*P* < 0.001 vs. LVS for all; Figure 4). The viability of LVS decreased approximately 0.8 log_10_, of Δ*oxyR* 2.8 log_10_, of Δ*katG* 3.0 log_10_, and of Δ*oxyR*/Δ*katG* 4.6 log_10_ CFU. The latter was significantly more susceptible than any of the other strains (*P* < 0.001; Figure 4).

In summary, all mutant strains displayed increased susceptibility to ONOO^−^ as compared to LVS, with the effect being similar for Δ*oxyR* and Δ*katG* and most pronounced for Δ*oxyR/*Δ*katG*.

### Gene expression

**Figure 5:**
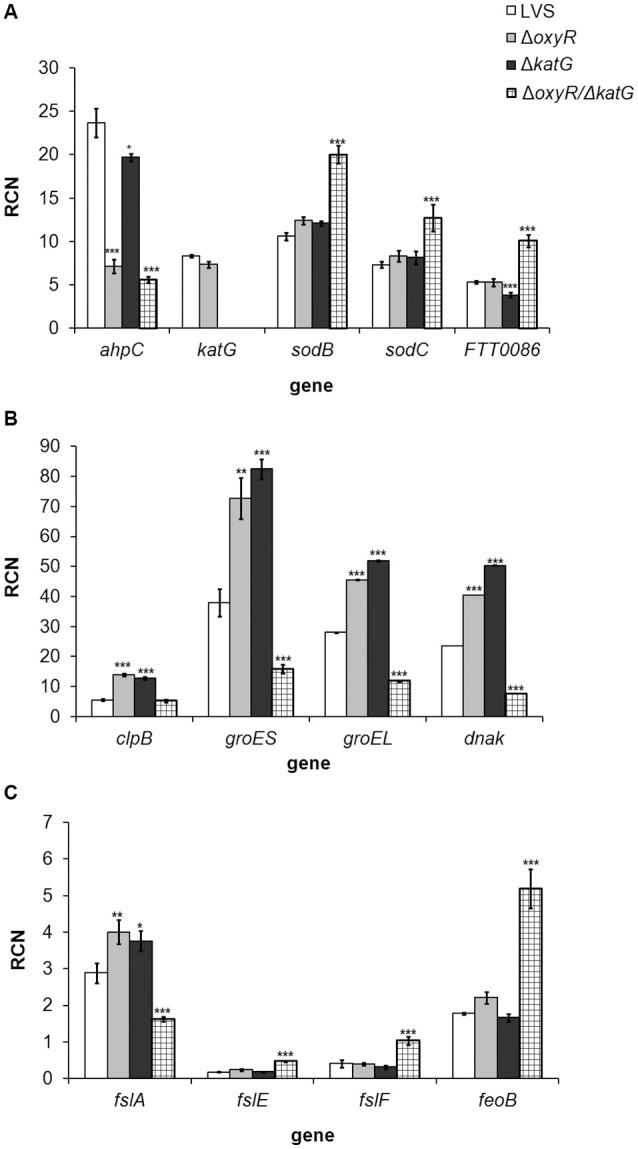
**Real-time PCR analysis of genes expressing (A)** antioxidant enzymes, **(B)** chaperones and **(C)** iron-related genes in *F. tularensis* strains cultivated in CDM for 10 h. Copy numbers of the respective gene in relation to the housekeeping gene *FTT0901* is shown (RCN). The bars represent the average from three separate experiments with triplicate samples in each and error bars represent the SEM. ^*^P < 0.05, ^**^P < 0.01, ^***^P < 0.001 vs. LVS.

Δ*oxyR/*Δ*katG* did not grow after the late logarithmic growth phase (Figure 1A), and we therefore found it of interest to explore the gene expression of the strains at 10 h, i.e., during the late logarithmic growth phase. The analysis was focused on genes expressing proteins influencing the oxidative stress response of the bacterium, such as antioxidant enzymes, chaperones and iron-related proteins. Genes found to be differentially expressed vs. LVS are shown in Figure 5. Of all genes examined, *ahpC* was the sole gene significantly repressed in Δ*oxyR* (*P* < 0.001; Figure 5A). A similar degree of repression, about 3-fold, was observed in Δ*oxyR/*Δ*katG*, which in addition, had a 1.5 to 2-fold increased expression of *sodB, sodC* and *FTT0086* (*P* < 0.001 for all genes; Figure 5A). *ahpC* was not repressed in Δ*katG* and as expected, *katG* transcripts were not detected in either Δ*katG* or in Δ*oxyR/*Δ*katG* (Figure 5A). All chaperone genes examined were upregulated 1.6 to 2.5-fold in Δ*oxyR* and Δ*katG* (*P* < 0.001 for all genes; Figure 5B). In contrast, these genes, except for *clpB*, were suppressed 2.4 to 3.1-fold in Δ*oxyR/*Δ*katG* (*P* < 0.001 for all genes).

*fslA*, the first gene of the siderophore operon, was slightly up-regulated in Δ*oxyR* and Δ*katG*, although only about 1.2-fold, whereas the other iron-related genes were expressed at similar levels as in LVS. In contrast, *fslA* was suppressed 1.8-fold in Δ*oxyR/*Δ*katG* and *fslE, fslF* and *feoB* were upregulated 2.5 to 2.9-fold (*P* < 0.001; Figure 5C).

In summary, the absence of OxyR resulted in a suppressed expression of *ahpC* and an up-regulated expression of genes encoding chaperone proteins. Except for *ahpC*, the expression profile of Δ*katG* was similar to Δ*oxyR*. In contrast, loss of both *oxyR* and *katG* changed the expression profile and low expression of chaperone-encoding genes was observed in Δ*oxyR/*Δ*katG*, together with high expression of antioxidant genes, except for *ahpC* and *katG*, and an altered expression of genes related to iron-uptake.

### Intracellular replication in BMDM

**Figure 6:**
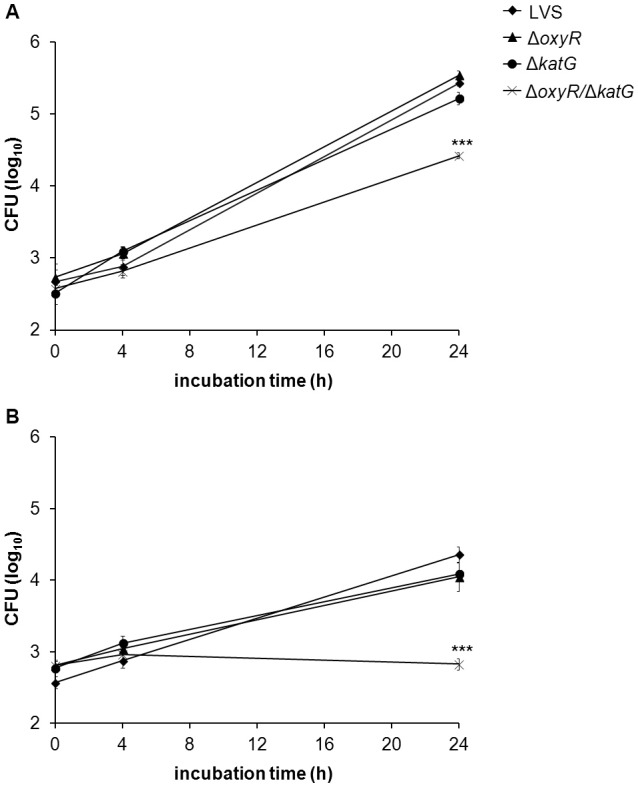
**Growth of LVS, Δ*oxyR*, Δ*katG*, and Δ*oxyR/*Δ*katG* in (A)** non-stimulated or **(B)** IFN-γ–stimulated BMDM. Monolayers of BMDM were infected with the various strains at an MOI of 30 and at indicated time points, intracellular bacteria were enumerated by lysis of the cultures and plating 10-fold serial dilutions on MC plates. The diagrams show one representative experiment, with the average of triplicate samples at each time point and treatment. Error bars represent the SEM. Similar results were observed in two additional experiments. ^***^P < 0.001 vs. LVS.

Based on the increased susceptibility to various ROS displayed by Δ*oxyR*, Δ*katG*, and Δ*oxyR*/Δ*katG*, it was of interest to test whether the strains were defective for replication in professional phagocytes. Non-stimulated or IFN-γ-stimulated BMDMs were infected with LVS, Δ*oxyR*, Δ*katG*, or Δ*oxyR*/Δ*katG* at an MOI of 30, and the viability of internalized bacteria was determined after 0 h, 4 h, and 24 h. In non-stimulated BMDM, LVS grew from approximately 2.5 log_10_ CFU to more than 5.0 log_10_ CFU within 24 h and also Δ*oxyR* and Δ*katG* grew to similar extent (Figure 6A). Δ*oxyR*/Δ*katG* grew in non-stimulated cells, but reached approximately 10-fold lower numbers compared to the other strains after 24 h (*P* < 0.001; Figure 6A).

IFN-γ-stimulation of BMDM prior to infection reduced the numbers of LVS, Δ*katG*, and Δ*oxyR* about 10-fold at 24 h vs. the numbers in non-stimulated cultures (*P* < 0.001; Figures 6A,B). There was no growth of Δ*oxyR/*Δ*katG* in IFN-γ-stimulated cultures and, thus, significantly lower bacterial numbers compared to non-stimulated cultures at 24 h (*P* < 0.001; Figures 6A,B) and vs. all the other strains exposed to IFN-γ (*P* < 0.001; Figure 6B).

Thus, the Δ*oxyR* and Δ*katG* mutants showed intact capacity of intracellular replication, whereas the Δ*oxyR/*Δ*katG* mutant showed impaired replication in BMDM, both in the presence and absence of IFN-γ.

### Virulence in mice

**Figure 7:**
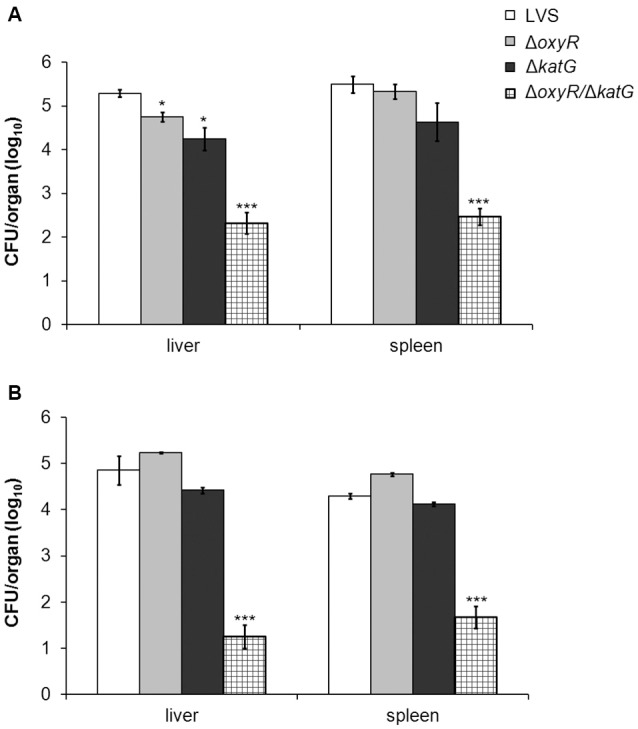
**Bacterial burden of mice at (A)** 3 and **(B)** 6 days after subcutaneous inoculation of 4 × 10^3^ CFU of either LVS, Δ*oxyR*, Δ*katG* or Δ*oxyR/*Δ*katG*. The average of four mice and SEM are shown. ^*^P < 0.05, ^***^P < 0.001 vs. LVS.

The virulence of LVS, Δ*oxyR*, Δ*katG*, and Δ*oxyR/*Δ*katG* was determined by subcutaneous infection of C57BL/6 mice with 4 × 10^3^ CFU/mouse, a non-lethal dose, and enumeration of viable bacteria in spleen and liver on day 3 and 6 of infection. Compared to LVS, there were lower numbers of both Δ*oxyR* and Δ*katG* on day 3 in the liver of the mice (*P* < 0.05; Figure 7A), whereas there were no differences between these strains in either the liver or spleen at the other time points (Figures 7A,B). Numbers of Δ*oxyR/*Δ*katG* in both organs were at least 100-fold lower vs. all other strains at both time points (*P* < 0.001). Thus, both Δ*oxyR* and Δ*katG* showed slight attenuation in mice, whereas Δ*oxyR/*Δ*katG* was highly attenuated.

## Discussion

*Francisella tularensis* is a versatile bacterium capable of surviving in many different hosts, vectors and in various cell types, including the normally bactericidal macrophages. Upon phagocytosis, *F. tularensis* is encased in a phagosome, a membrane-bound compartment designed for the annihilation of phagocytosed microbes, which is rich in antimicrobial molecules, such as reactive oxygen and nitrogen species. Although *F. tularensis* only transiently resides in this compartment, it must still muster defenses against highly reactive species in order to survive and escape to the cytosol, where it proceeds to replicate. By entering the cytosol, *F. tularensis* gains access to a nutrient-rich, protected niche in which it multiplies. As survival and replication in the intracellular niche is essential for the life cycle of *F. tularensis*, a thorough understanding of how the bacterium survives intracellularly is essential to fully grasp its defense mechanisms against oxidative stress. To this end, the study focused on understanding the interplay between catalase and OxyR, the latter being important for the expression of several antioxidant enzymes, in the defense against ROS and their impact on the survival of the bacterium in professional phagocytes.

To investigate if OxyR is involved in the oxidative stress response of LVS, we constructed an in-frame deletion of *oxyR*. A similar investigation has been performed recently by Ma et al. which studied the role of OxyR in LVS (Ma et al., 2016). It was found that OxyR controlled transcription of *katG* and the findings agree with the reduced catalase activity of Δ*oxyR* observed in the present study. Nevertheless, our study revealed that even in the absence of OxyR, there was still prominent catalase activity. Overall, it appears that OxyR, as expected, regulates *katG* in the LVS strain, however, the regulation does not completely abolish its expression as is the case observed for various other bacterial species, e.g., *E. coli* (Michán et al., 1999), *Salmonella enterica* (Morgan et al., 1986), *Haemophilus influenza* (Whitby et al., 2012), or *Moraxella catarrhalis* (Hoopman et al., 2011). In both the present study, and in the previous study, it was observed that the lack of OxyR led to marked suppression of *ahpC2* (Ma et al., 2016). In addition Ma et al. demonstrated suppressed expression of both *katG* and *sodB* in Δ*oxyR* by real-time PCR and demonstrated that OxyR binds to the upstream promoter regions of each gene. In contrast, there was no down-regulation of *katG* or *sodB* observed in the present study. Likely, this is a consequence of the rapid on/off switch of the promoter binding capacity of OxyR in response to the oxidative levels in the bacteria leading to a limited window when elevated mRNA levels can be detected (Wei et al., 2012).

Besides antioxidant genes, our study revealed an aberrant expression of genes encoding chaperone proteins of the mutants. Such proteins are induced in response to various stresses, including oxidative stress (Hartl et al., 2011). Thus, the induced expression of these genes in Δ*oxyR* and in Δ*katG*, also observed by Ma et al. (2016), likely is a reflection of oxidative stress encountered by the mutants. The chaperone network likely helps the bacterium to handle this stress through unfolding and/or degradation of mis-folded/damaged proteins. The reason behind the suppressed expression of multiple chaperone genes in Δ*oxyR/*Δ*katG* is obscure, but should lead to an accumulation of damaged or mis-folded proteins and may explain why it was so impaired for growth in broth. The intact growth of Δ*oxyR/*Δ*katG* under microaerobic conditions likely reflects that reduced levels of ROS are formed and therefore that antioxidant defenses are less important. The aberrant expression of genes related to iron-uptake did not result in a skewed iron content of Δ*oxyR/*Δ*katG* (data not shown) and it is therefore not obvious that this would influence the susceptibility of the strain to various ROS.

The *F. tularensis ahpC2* gene is divergently transcribed from the *oxyR* promoter, a feature commonly seen for genes transcriptionally regulated by OxyR (Hahn et al., 2002; Maddocks and Oyston, 2008). AhpC belongs to the peroxiredoxin family, which is ubiquitously found in nature (Rhee et al., 2005) and is known to be involved in defenses against peroxides in *E. coli* (Storz et al., 1989), and both peroxides and peroxynitrite in, e.g., *Salmonella typhimurium* (Bryk et al., 2000), and in the defense against superoxide and peroxynitrite in the virulent SCHU S4 strain of *F. tularensis* subsp. *tularensis* (Binesse et al., 2015). In agreement with this, and in view of the reduced expression of AhpC in Δ*oxyR*, this mutant was also highly susceptible to ONOO^−^. Δ*katG* was as susceptible as Δ*oxyR* to ONOO^−^ and in view of the substantial catalase activity remaining in Δ*oxyR*, this result implies that the function of catalase overlaps with other OxyR-regulated detoxifying mechanisms, presumably AhpC, to protect against ONOO^−^. Further corroborating the importance of AhpC and catalase was the failure to generate a *katG* and *ahpC* double deletion mutant and even an *ahpC* mutant in LVS. Hence, AhpC seems indispensable to LVS, which is in stark contrast to SCHU S4, where deletion of *ahpC* resulted in only slight attenuation (Binesse et al., 2015). This indicates that there is a disparity regarding the importance of the enzyme between the SCHU S4 and LVS strains, possibly a factor that to some extent explains the difference in virulence between the strains, since it implies that the detoxifying mechanisms of SCHU S4 are much more elaborate. Nevertheless, as for LVS, it has not been possible to generate a *katG* and *ahpC* double deletion mutant of SCHU S4 (Kadzhaev et al., 2009; Binesse et al., 2015). Collectively, this indicates that the mechanisms of protection conferred by these enzymes may be overlapping and the lack of both is detrimental to the survival of both LVS and highly virulent *F. tularensis* strains.

Based on the failure to generate a *katG* and *ahpC* double deletion mutant and the marked suppression of *ahpC* in the Δ*oxyR* mutant, we hypothesized that the absence of OxyR together with the absence of catalase would severely disarm the capability of the bacterium to handle ROS. Indeed, we observed that the Δ*oxyR*/Δ*katG* mutant was hyper-susceptible to H_2_O_2_, ONOO^−^, and ${\text{O}}_{2}^{-}$; much more so than either Δ*oxyR* or Δ*katG*. Collectively, the results demonstrate that the roles of OxyR-regulated antioxidant enzymes and catalase overlap to protect LVS against various ROS. We find it likely that the reduced activity of catalase and expression of *ahpC* observed in *oxyR* contributed to the increased susceptibility of the mutant to H_2_O_2_, ${\text{O}}_{2}^{-}$, and ONOO^−^ through the increase of both Fenton-mediated toxicity and direct ${\text{O}}_{2}^{-}$- and ONOO^−^-mediated damage. We further suggest that the reduced levels of AhpC together with the lack of catalase in the Δ*oxyR/*Δ*katG* strain, despite an increased expression of *sodB, sodC* and *FTT0086*, resulted in enhanced Fenton-mediated toxicity and ONOO^−^-mediated damage, which likely account for the extreme susceptibility of the double mutant to ${\text{O}}_{2}^{-}$, H_2_O_2_, and ONOO^−^. Our findings concur with those of Ma et al. (2016), and, in addition, demonstrate that the combined activity of catalase and OxyR-regulated detoxifying mechanisms are critical for ROS detoxification by *F. tularensis*.

Despite the enhanced susceptibility of both Δ*oxyR* and Δ*katG* to various ROS, the strains replicated as efficiently as LVS in mouse BMDM, but importantly, the capacity to replicate in professional phagocytes required either OxyR or catalase, since Δ*oxyR*/Δ*katG* failed to replicate. IFN-γ-activation of BMDM restricted growth of LVS, Δ*katG*, and Δ*oxyR* to a similar degree and completely blocked the growth of Δ*oxyR/*Δ*katG*. The majority of *F. tularensis* LVS escapes the phagosome of IFN-γ-activated macrophages (Lindgren et al., 2004), but the mechanism of growth inhibition appears to vary depending on the cell model used (Edwards et al., 2010). IFN-γ-mediated inhibition of intracellular growth of *F. novicida* is dependent on the expression of IRGB10 and various guanylate-binding proteins (Meunier et al., 2015; Man et al., 2016), however, the role of this pathway is unknown for other *F. tularensis* species.

Our results reveal elaborate interconnecting roles between OxyR-regulated ROS-detoxifying mechanisms and catalase and demonstrate that either needs to be intact for the bacterium to thrive in professional phagocytes. The roles of the anti-oxidative mechanisms could be to protect the bacterium from direct damage by various ROS, such as ONOO^−^, which has been demonstrated to be crucial for killing of *F. tularensis* in peritoneal cells (Lindgren et al., 2005). Alternatively, or additionally, the antioxidants may restrict macrophage activation through their ability to preserve phosphatase activity required for kinase signaling and proinflammatory cytokine production (Melillo et al., 2010).

Our finding that Δ*oxyR* replicated as efficiently as LVS in BMDM is in contrast to findings in a previous study, which reported that an *oxyR* mutant of LVS was markedly impaired with regard to escape from the phagosome, replication in professional phagocytes, and virulence in the mouse model (Ma et al., 2016). Notably, the LVS strain used by Ma et al. replicated less than 10-fold during 24 h in C57BL/6 BMDM, whereas the LVS strain used in the present study replicated about 500-fold. Isolates of LVS with different virulence are used in the research community (Griffin et al., 2015) and the distinct differences in the intracellular growth of these two LVS strains are additional examples of such distinct phenotypes. The phenotypic differences between the two LVS strains likely explain the discrepant findings of the two studies. The observation in the present study of the intact growth of the single mutants in BMDM was corroborated by findings *in vivo*, since the Δ*oxy*R and Δ*kat*G mutants showed essentially intact growth in organs of mice, whereas Δ*oxy*R/Δ*kat*G hardly grew at all. Despite their effective growth in the organs, a previous study demonstrated a more distinct growth defect of the Δ*kat*G mutant, most likely because a 100-fold higher dose was given (Lindgren et al., 2007). Moreover, by the intranasal route, Δ*oxy*R was demonstrated to be moderately attenuated (Ma et al.). Based on these collective findings, it can be concluded that both OxyR and KatG contribute to the virulence of *F. tularensis* LVS and that the concomitant loss is detrimental to the virulence of the bacterium.

Altogether, the results presented in this study clearly demonstrate the mutual importance of catalase and OxyR for a robust oxidative stress defense system and that either of these systems is vital for the intracellular replication of *F. tularensis* and for its virulence.

## Author contributions

Conceived and designed the experiments: AS, MH, and HL. Performed the experiments: MH, HL, and GB. Analyzed the data: AS, MH, HL, and GB. Wrote the paper: HL, MH, and AS.

### Conflict of interest statement

The authors declare that the research was conducted in the absence of any commercial or financial relationships that could be construed as a potential conflict of interest.

## References

[B1] Andrews S. C. Robinson A. K. Rodríguez-Quiñones F. (2003). Bacterial iron homeostasis. FEMS Microbiol. Rev. 27, 215–237. 10.1016/S0168-6445(03)00055-X 12829269

[B2] Anthony L. S. Morrissey P. J. Nano F. E. (1992). Growth inhibition of *Francisella tularensis* live vaccine strain by IFN-gamma-activated macrophages is mediated by reactive nitrogen intermediates derived from L-arginine metabolism. J. Immunol. 148, 1829–1834. 1541823

[B3] Bakshi C. S. Malik M. Regan K. Melendez J. A. Metzger D. W. Pavlov V. M.. (2006). Superoxide dismutase B gene (sodB)-deficient mutants of *Francisella tularensis* demonstrate hypersensitivity to oxidative stress and attenuated virulence. J. Bacteriol. 188, 6443–6448. 10.1128/JB.00266-06 16923916 PMC1595384

[B4] Betteridge D. J. (2000). What is oxidative stress? Metab. Clin. Exp. 49, 3–8. 10.1016/S0026-0495(00)80077-3 10693912

[B5] Binesse J. Lindgren H. Lindgren L. Conlan W. Sjöstedt A. (2015). Roles of reactive oxygen species-degrading enzymes of *Francisella tularensis* SCHU S4. Infect. Immun. 83, 2255–2263. 10.1128/IAI.02488-14 25802058 PMC4432764

[B6] Bröms J. E. Lavander M. Meyer L. Sjöstedt A. (2011). IglG and IglI of the *Francisella pathogenicity* island are important virulence determinants of *Francisella tularensis* LVS. Infect. Immun. 79, 3683–3696. 10.1128/IAI.01344-10 21690239 PMC3165494

[B7] Bröms J. E. Lavander M. Sjöstedt A. (2009). A conserved alpha-helix essential for a type VI secretion-like system of *Francisella tularensis*. J. Bacteriol. 191, 2431–2446. 10.1128/JB.01759-08 19201795 PMC2668417

[B8] Bröms J. E. Sjöstedt A. Lavander M. (2010). The role of the *Francisella tularensis* pathogenicity island in type VI secretion, intracellular survival, and modulation of host cell signaling. Front. Microbiol. 1:136. 10.3389/fmicb.2010.00136 21687753 PMC3109350

[B9] Bryk R. Griffin P. Nathan C. (2000). Peroxynitrite reductase activity of bacterial peroxiredoxins. Nature 407, 211–215. 10.1038/35025109 11001062

[B10] Chong A. Celli J. (2010). The *Francisella intracellular* life cycle: toward molecular mechanisms of intracellular survival and proliferation. Front. Microbiol. 1:138. 10.3389/fmicb.2010.00138 21687806 PMC3109316

[B11] Conlan J. W. (2011). Tularemia vaccines: recent developments and remaining hurdles. Future Microbiol. 6, 391–405. 10.2217/fmb.11.22 21526941

[B12] Edwards J. A. Rockx-Brouwer D. Nair V. Celli J. (2010). Restricted cytosolic growth of *Francisella tularensis* subsp. *tularensis* by IFN-gamma activation of macrophages. Microbiology 156, 327–339. 10.1099/mic.0.031716-0 19926654 PMC2890092

[B13] Farr S. B. Kogoma T. (1991). Oxidative stress responses in *Escherichia coli* and *Salmonella typhimurium*. Microbiol. Rev. 55, 561–585. 1779927 10.1128/mr.55.4.561-585.1991 PMC372838

[B14] Flannagan R. S. Cosio G. Grinstein S. (2009). Antimicrobial mechanisms of phagocytes and bacterial evasion strategies. Nat. Rev. Microbiol. 7, 355–366. 10.1038/nrmicro2128 19369951

[B15] Fridovich I. (1998). Oxygen toxicity: a radical explanation. J. Exp. Biol. 201, 1203–1209. 9510531 10.1242/jeb.201.8.1203

[B16] Gavrilin M. A. Bouakl I. J. Knatz N. L. Duncan M. D. Hall M. W. Gunn J. S.. (2006). Internalization and phagosome escape required for *Francisella* to induce human monocyte IL-1β processing and release. Proc. Natl. Acad. Sci. U.S.A. 103, 141–146. 10.1073/pnas.0504271103 16373510 PMC1324976

[B17] Golovliov I. Sjöstedt A. Mokrievich A. Pavlov V. (2003). A method for allelic replacement in *Francisella tularensis*. FEMS Microbiol. Lett. 222, 273–280. 10.1016/S0378-1097(03)00313-6 12770718

[B18] Griffin A. J. Crane D. D. Wehrly T. D. Bosio C. M. (2015). Successful protection against tularemia in C57BL/6 mice is correlated with expansion of *Francisella tularensis*-specific effector T cells. Clin. Vaccine Immunol. 22, 119–128. 10.1128/CVI.00648-14 25410207 PMC4278928

[B19] Hahn J. S. Oh S. Y. Roe J. H. (2002). Role of OxyR as a peroxide-sensing positive regulator in *Streptomyces coelicolor* A3(2). J. Bacteriol. 184, 5214–5222. 10.1128/JB.184.19.5214-5222.2002 12218006 PMC137946

[B20] Hartl F. U. Bracher A. Hayer-Hartl M. (2011). Molecular chaperones in protein folding and proteostasis. Nature 475, 324–332. 10.1038/nature10317 21776078

[B21] Hassan H. M. Fridovich I. (1979). Paraquat and *Escherichia coli*. Mechanism of production of extracellular superoxide radical. J. Biol. Chem. 254, 10846–10852. 227855

[B22] Hogg N. Darley-Usmar V. M. Wilson M. T. Moncada S. (1992). Production of hydroxyl radicals from the simultaneous generation of superoxide and nitric oxide. Biochem. J. 281, 419–424. 10.1042/bj2810419 1310595 PMC1130701

[B23] Honn M. Lindgren H. Sjöstedt A. (2012). The role of MglA for adaptation to oxidative stress of *Francisella tularensis* LVS. BMC Microbiol. 12:14. 10.1186/1471-2180-12-14 22264342 PMC3305382

[B24] Hoopman T. C. Liu W. Joslin S. N. Pybus C. Brautigam C. A. Hansen E. J. (2011). Identification of gene products involved in the oxidative stress response of *Moraxella catarrhalis*. Infect. Immun. 79, 745–755. 10.1128/IAI.01060-10 21098105 PMC3028835

[B25] Kadzhaev K. Zingmark C. Golovliov I. Bolanowski M. Shen H. Conlan W.. (2009). Identification of genes contributing to the virulence of *Francisella tularensis* SCHU S4 in a mouse intradermal infection model. PLoS ONE 4:e5463. 10.1371/journal.pone.0005463 19424499 PMC2675058

[B26] Lindgren H. Honn M. Golovlev I. Kadzhaev K. Conlan W. Sjöstedt A. (2009). The 58-kilodalton major virulence factor of *Francisella tularensis* is required for efficient utilization of iron. Infect. Immun. 77, 4429–4436. 10.1128/IAI.00702-09 19651867 PMC2747937

[B27] Lindgren H. Shen H. Zingmark C. Golovliov I. Conlan W. Sjöstedt A. (2007). Resistance of *Francisella tularensis* strains against reactive nitrogen and oxygen species with special reference to the role of KatG. Infect. Immun. 75, 1303–1309. 10.1128/IAI.01717-06 17210667 PMC1828546

[B28] Lindgren H. Stenman L. Tärnvik A. Sjöstedt A. (2005). The contribution of reactive nitrogen and oxygen species to the killing of *Francisella tularensis* LVS by murine macrophages. Microbes Infect. 7, 467–475. 10.1016/j.micinf.2004.11.020 15788155

[B29] Lindgren H. Stenmark S. Chen W. Tärnvik A. Sjöstedt A. (2004). Distinct roles of reactive nitrogen and oxygen species to control infection with the facultative intracellular bacterium Francisella tularensis. Infect. Immun. 72, 7172–7182. 10.1128/IAI.72.12.7172-7182.2004 15557642 PMC529105

[B30] Ma Z. Russo V. C. Rabadi S. M. Jen Y. Catlett S. V. Bakshi C. S.. (2016). Elucidation of a mechanism of oxidative stress regulation in *Francisella tularensis* live vaccine strain. Mol. Microbiol. 101, 856–878. 10.1111/mmi.13426 27205902 PMC5134674

[B31] Maddocks S. E. Oyston P. C. (2008). Structure and function of the LysR-type transcriptional regulator (LTTR) family proteins. Microbiology 154, 3609–3623. 10.1099/mic.0.2008/022772-0 19047729

[B32] Man S. M. Karki R. Sasai M. Place D. E. Kesavardhana S. Temirov J.. (2016). IRGB10 liberates bacterial ligands for sensing by the AIM2 and Caspase-11-NLRP3 inflammasomes. Cell 167, 382–396.e17. 10.1016/j.cell.2016.09.012 27693356 PMC5074697

[B33] Melillo A. A. Bakshi C. S. Melendez J. A. (2010). *Francisella tularensis* antioxidants harness reactive oxygen species to restrict macrophage signaling and cytokine production. J. Biol. Chem. 285, 27553–27560. 10.1074/jbc.M110.144394 20558723 PMC2934622

[B34] Melillo A. A. Mahawar M. Sellati T. J. Malik M. Metzger D. W. Melendez J. A.. (2009). Identification of *Francisella tularensis* live vaccine strain CuZn superoxide dismutase as critical for resistance to extracellularly generated reactive oxygen species. J. Bacteriol. 191, 6447–6456. 10.1128/JB.00534-09 19684141 PMC2753026

[B35] Meunier E. Wallet P. Dreier R. F. Costanzo S. Anton L. Rühl S.. (2015). Guanylate-binding proteins promote activation of the AIM2 inflammasome during infection with Francisella novicida. Nat. Immunol. 16, 476–484. 10.1038/ni.3119 25774716 PMC4568307

[B36] Michán C. Manchado M. Dorado G. Pueyo C. (1999). *In vivo* transcription of the *Escherichia coli* oxyR regulon as a function of growth phase and in response to oxidative stress. J. Bacteriol. 181, 2759–2764. 10217765 10.1128/jb.181.9.2759-2764.1999 PMC93716

[B37] Morgan R. W. Christman M. F. Jacobson F. S. Storz G. Ames B. N. (1986). Hydrogen peroxide-inducible proteins in *Salmonella typhimurium* overlap with heat shock and other stress proteins. Proc. Natl. Acad. Sci. U.S.A. 83, 8059–8063. 10.1073/pnas.83.21.8059 3534881 PMC386866

[B38] Murphy M. P. Packer M. A. Scarlett J. L. Martin S. W. (1998). Peroxynitrite: a biologically significant oxidant. Gen. Pharmacol. 31, 179–186. 10.1016/S0306-3623(97)00418-7 9688457

[B39] Oyston P. C. Sjöstedt A. Titball R. W. (2004). Tularaemia: bioterrorism defence renews interest in Francisella tularensis. Nat. Rev. Microbiol. 2, 967–978. 10.1038/nrmicro1045 15550942

[B40] Polsinelli T. Meltzer M. S. Fortier A. H. (1994). Nitric oxide-independent killing of *Francisella tularensis* by IFN-gamma-stimulated murine alveolar macrophages. J. Immunol. 153, 1238–1245. 8027551

[B41] Pomposiello P. J. Demple B. (2001). Redox-operated genetic switches: the SoxR and OxyR transcription factors. Trends Biotechnol. 19, 109–114. 10.1016/S0167-7799(00)01542-0 11179804

[B42] Rhee S. G. Chae H. Z. Kim K. (2005). Peroxiredoxins: a historical overview and speculative preview of novel mechanisms and emerging concepts in cell signaling. Free Radic. Biol. Med. 38, 1543–1552. 10.1016/j.freeradbiomed.2005.02.026 15917183

[B43] Schaible U. E. Kaufmann S. H. (2004). Iron and microbial infection. Nat. Rev. Microbiol. 2, 946–953. 10.1038/nrmicro1046 15550940

[B44] Sjöstedt A. (2006). Intracellular survival mechanisms of Francisella tularensis, a stealth pathogen. Microbes Infect. 8, 561–567. 10.1016/j.micinf.2005.08.001 16239121

[B45] Storz G. Jacobson F. S. Tartaglia L. A. Morgan R. W. Silveira L. A. Ames B. N. (1989). An alkyl hydroperoxide reductase induced by oxidative stress in *Salmonella typhimurium* and *Escherichia coli*: genetic characterization and cloning of ahp. J. Bacteriol. 171, 2049–2055. 10.1128/jb.171.4.2049-2055.1989 2649484 PMC209856

[B46] Troxell B. Hassan H. M. (2013). Transcriptional regulation by Ferric Uptake Regulator (Fur) in pathogenic bacteria. Front. Cell. Infect. Microbiol. 3:59. 10.3389/fcimb.2013.00059 24106689 PMC3788343

[B47] Wei Q. Minh P. N. Dötsch A. Hildebrand F. Panmanee W. Elfarash A.. (2012). Global regulation of gene expression by OxyR in an important human opportunistic pathogen. Nucleic Acids Res. 40, 4320–4333. 10.1093/nar/gks017 22275523 PMC3378865

[B48] Whitby P. W. Morton D. J. Vanwagoner T. M. Seale T. W. Cole B. K. Mussa H. J.. (2012). *Haemophilus influenzae* OxyR: characterization of its regulation, regulon and role in fitness. PLoS ONE 7:e50588. 10.1371/journal.pone.0050588 23226321 PMC3511568

[B49] Zheng M. Aslund F. Storz G. (1998). Activation of the OxyR transcription factor by reversible disulfide bond formation. Science 279, 1718–1721. 10.1126/science.279.5357.1718 9497290

[B50] Zheng M. Doan B. Schneider T. D. Storz G. (1999). OxyR and SoxRS regulation of fur. J. Bacteriol. 181, 4639–4643. 10419964 10.1128/jb.181.15.4639-4643.1999 PMC103597

